# Shortening migration by 4500 km does not affect nesting phenology or increase nest success for black brant (*Branta bernicla nigricans*) breeding in Arctic and subarctic Alaska

**DOI:** 10.1186/s40462-025-00530-z

**Published:** 2025-03-25

**Authors:** Toshio D. Matsuoka, Vijay P. Patil, Jerry W. Hupp, Alan G. Leach, John A. Reed, James S. Sedinger, David H. Ward

**Affiliations:** 1 U.S. Geological Survey, Alaska Science Center, 4210 University Drive, Anchorage, AK 99508 USA; 2 Department of Natural Resources and Environmental Science, University of Nevada Reno, 1664 N. Virginia Street, Reno, NV 89557 USA

**Keywords:** Brant, Migration, Geolocators, Migratory network, Nest survival, Alaska, Arctic, Subarctic

## Abstract

**Background:**

Since the 1980s, Pacific Black Brant (*Branta bernicla nigricans*, hereafter brant) have shifted their winter distribution northward from Mexico to Alaska (approximately 4500 km) with changes in climate. Alongside this shift, the primary breeding population of brant has declined. To understand the population-level implications of the changing migration strategy of brant, it is important to connect movement and demographic data. Our objectives were to calculate migratory connectivity, a measure of spatial and temporal overlap during the non-breeding period, for Arctic and subarctic breeding populations of brant, and to determine if variation in migration strategies affected nesting phenology and nest survival.

**Methods:**

We derived a migratory network using light-level geolocator migration tracks from an Arctic site (Colville River Delta) and a subarctic site (Tutakoke River) in Alaska. Using this network, we quantified the migratory connectivity of the two populations during the winter. We also compared nest success rates among brant that used different combinations of winter sites and breeding sites.

**Results:**

The two breeding populations were well mixed during the winter, as indicated by a migratory connectivity score close to 0 (− 0.06) at the primary wintering sites of Izembek Lagoon, Alaska (n = 11 brant) and Baja California, Mexico (n = 48). However, Arctic birds were more likely to migrate the shorter distance to Izembek (transition probability = 0.24) compared to subarctic birds (transition probability = 0.09). Nest survival for both breeding populations was relatively high (0.88–0.92), and we did not detect an effect of wintering site on nest success the following year.

**Conclusions:**

Nest survival of brant did not differ among brant that used wintering sites despite a 4500 km difference in migration distances. Our results also suggested that the growing Arctic breeding population is unlikely to compensate for declines in the larger breeding population of brant in the subarctic. However, this study took place in 2011–2014 and wintering at Izembek Lagoon may have greater implications for reproductive success under future climate conditions.

## Background

Avian migrants are faced with numerous challenges during their annual flights between breeding and wintering areas. Migration allows birds that breed at high latitudes to escape harsh winter conditions and periods of low food availability, but these long-distance flights, are energetically costly [1], entail substantial navigational demands [2], and expose birds to hazardous weather conditions [3]. Consequently, the timing and pathways of annual migrations can affect components of individual fitness, including mortality risk and the likelihood of reproductive success [4–6], which can, in turn, influence population trajectories [7–9].

Many birds that breed at high latitudes have a broad winter distribution, encompassing a variety of environmental conditions and migration strategies [1, 3, 10, 11]. The variation in weather conditions and food availability across the winter distribution can result in a range of energetic costs for migratory birds [12]. If food availability is similar among winter locations, wintering at lower latitudes should be less energetically demanding because warmer weather conditions reduce the expense of thermoregulation [13]. However, these lower latitude sites are often thousands of kilometers away from the breeding grounds, requiring substantial energy for migration [1, 14]. In contrast, wintering at high latitudes offers shorter flight paths, allowing birds to spend less energy on migration [12], but is often coupled with colder temperatures and harsher weather conditions that have energetic costs [15–17]. Such variation produces a range of effects on avian migrants, but these effects may be changing due to the ecological impacts of climate change, which is causing more rapid warming at high latitudes and increasing phenological asynchrony across the migratory range of many species [18, 19].

Long-term trends towards warmer temperatures, reduced snow and ice cover [20, 21], and increased access to food resources [22] at high latitudes have allowed many waterfowl populations to shorten their fall migrations [21]. An extreme example of this is the Black Brant (*Branta bernicla nigricans*), a small-bodied migratory goose that nests in Arctic (> 66° North Latitude) and subarctic coastal habitats in Alaska, Canada, and Russia. Black brant comprise the vast majority of brant in the Pacific Flyway of North America and are sometimes also referred to as Pacific brant [23]. Black brant (hereafter brant) have historically wintered at extensive eelgrass beds along the Pacific coast of the continental United States and Baja California, Mexico, but have been shifting their winter distribution northward since the early 1980s [22]. Since 2011, more than 25% of the population has spent the winter on the Alaska Peninsula within the Izembek National Wildlife Refuge (55.27° N, 162.91° W; hereafter Izembek), 4,500 km north of their main wintering sites in Baja California, Mexico (hereafter Mexico). [10, 24]. Izembek plays a unique role in the brant life cycle because it is the fall staging location for nearly the entire population [22]. Brant use this location to feed on eelgrass (*Zostera* spp.), their primary food outside the breeding season [23, 25], and in recent decades warmer winters and reduced shore-fast ice cover have allowed year-round access to intertidal eelgrass beds [23]. Brant may be more responsive to climate-driven habitat changes across their migratory range compared to other geese due to their small body size and capital breeding strategy [26].

Wintering at Izembek could benefit brant and increase their reproductive success if it allows them to take advantage of the ongoing trend towards earlier spring green-up and longer growing seasons at high latitudes [27] by facilitating an earlier return to breeding areas and earlier nest initiation [28]. Earlier arrival to breeding areas allows for earlier nesting, faster growth, and larger size at the fledging of goslings, all of which are associated with higher gosling survival and likelihood of recruitment into the breeding population [29–32]. However, wintering at Izembek has high risks of unexpected cold winters, extreme icing conditions, and associated annual variation in eelgrass abundance [17].

There is strong motivation to understand changes in the wintering distribution of brant because their primary breeding populations in Alaska show diverging population trends, and because the metapopulation structure connecting these breeding populations remains poorly understood [23, 33–36]. During the breeding season, over 75% of brant were historically found on the Yukon-Kuskokwim Delta in subarctic Alaska [37]. Since the 1990s, however, nest numbers on the Yukon-Kuskokwim Delta have declined nearly 60% (1992–2017) [38], in part due to nest predation by Arctic foxes (*Alopex lagopus*) and reduced availability of food in grazing lawns [39, 40]. The largest breeding concentration outside the subarctic Yukon-Kuskokwim Delta occurs 1200 km further north on the Arctic Coastal Plain of Alaska, an area with abundant high-quality brood-rearing habitat [41–43]. The Arctic breeding population in Alaska has increased by 580% since the early 1990s, but it remains six times smaller than the subarctic population [33]. We do not know the extent to which the two populations mix outside of the breeding season, which could allow Arctic recruitment to partially offset subarctic losses. It is also unclear whether breeding population dynamics have been affected by increased overwintering at Izembek. The winter population at Izembek is thought to consist primarily of brant from Arctic breeding sites [22] but could also include failed and nonbreeding birds from both breeding locations that lack sufficient energy reserves necessary to migrate farther south. Therefore, it is not clear if wintering at Izembek represents an adaptive response to environmental change.

We deployed light-level geolocator devices on nesting brant at Arctic and subarctic breeding areas in Alaska to address three questions: (1) Do Arctic-breeding brant have a different migration strategy than subarctic-breeding brant, regarding their winter distribution and migration chronology? (2) Is wintering at Izembek instead of historical wintering areas associated with increased or decreased nest success, an important component of individual fitness? (3) Do the migration strategies of Arctic and subarctic birds help explain their differing population trajectories? To address these questions, our first objective was to estimate migratory connectivity, a measure of spatial overlap in migratory pathways, between Arctic and subarctic breeding populations. Second, we sought to measure the strength of each breeding ground's connections to known eelgrass beds that represented potential winter habitat along the west coast of North America. Third, we assessed temporal differences in the migratory movements of brant from each breeding area. Finally, we used light-level geolocators to estimate nest success rates and link them to the migratory movements of individual brant during the year prior to nesting.

## Methods

We used light-level geolocators (hereafter, geolocators) to estimate the migratory movements of brant from Arctic and subarctic breeding populations in Alaska. Geolocators, which use ambient light and an internal clock to estimate locations [44], have been widely used to model movements of migratory bird species throughout the annual cycle and have little to no effect on survival or behavior for waterfowl and other bird species of a similar size [45, 46]. As a lightweight technology with low energy requirements, geolocators can be deployed on plastic tarsal bands and record migratory movements for a full year or more while imposing minimal energetic costs on the animal. Additionally, if they are deployed before the start of fall migration and retrieved the following summer, light data from geolocators can be used to identify the start and end of the incubation period during the subsequent breeding season, which can be used to estimate daily nest survival and the probability of nest success [47, 48].

**Fig. 1:**
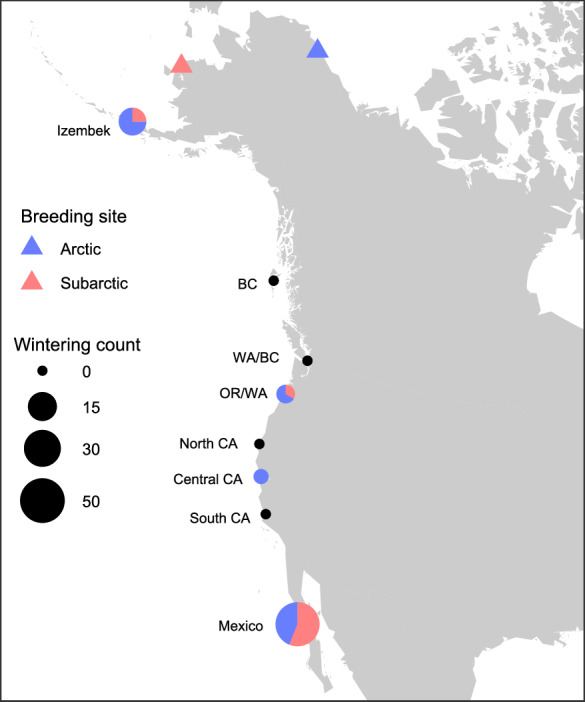
Breeding and wintering sites of geolocator-tagged Pacific black brant* (Branta bernicla nigricans*) in North America. Brant were tagged with geolocators at a subarctic breeding site (Tutakoke River, Yukon Kuskokwim Delta National Wildlife Refuge; 61.3° N, 165.6° W) and an Arctic breeding site (Colville River Delta; 70.42° N, 150.38° W) in Alaska, USA. The size of circles indicates the total number of geolocator-tagged brant that were tracked to each wintering site (n = 63). The colors of the pie-chart slices indicate the proportion of geolocator-tagged birds from each breeding location. Black wintering site points indicate that no birds with geolocators were present for the winter period (WA = Washington, BC = British Columbia, CA = California, Mexico = Baja California, Mexico)

We deployed geolocators on breeding adult female brant at a breeding colony in the Arctic at the Colville River Delta (70.42° N, 150.38° W), Alaska, and in the subarctic at the Tutakoke River (61.3° N, 165.6° W), Yukon Delta National Wildlife Refuge (Fig. 1) [24]. Most Arctic deployments occurred during annual late summer brood drives using a corral trap. The captured sample from the Arctic consisted almost exclusively of family groups with < 0.01% second year birds, and > 99% of captured females had brood patches, so tagged brant were likely to have nested and successfully hatched a clutch the year they were captured [49]. Breeding females in the subarctic were captured on nests in late incubation using a bow trap [50, 51]. Each bird was fitted with standard metal and alphanumeric inscribed colored plastic tarsal bands [29]. For a subset, we glued and zip-tied a 2 g geolocator onto the plastic tarsal band. Brant have high return rates to their natal breeding grounds [35] and we retrieved geolocators primarily by capturing birds in subsequent years of brood drives or nest-trapping. Geolocators were also retrieved from tagged birds from both breeding populations if they were shot during the hunting season.

### Geolocator analysis

We deployed four types of geolocators (Mk18, British Antarctic Survey, Cambridge, United Kingdom; W65 and C65K, Migrate Technology Inc. Cambridge, United Kingdom; MK5090, Lotek UK Ltd. [formerly Biotrack Ltd.], Wareham, United Kingdom) with differing data collection parameters but used a single calibration protocol and analytical framework for all tags. The geolocators measured light intensity every minute and logged the maximum measurement at two- (MK5090) or five- (W65, C65K, and Mk18) minute intervals. The Mk18 geolocators recorded light levels ranging from 0 to 64 lx, only capturing large changes in ambient light levels, such as dawn and dusk. However, the other tag types did not truncate light levels and could detect a wider range in ambient light levels. Geolocators were linearly corrected for desynchronization between the internal clock and Coordinated Universal Time using software provided by the tag manufacturer. Once adjusted, we analyzed all geolocator data in R version 4.3.1 [52] using package *TwGeos* 0.1.2 [53] to annotate twilights and *FLightR* 0.5.4 [54] to estimate locations. This included processing the data from each tag in six main steps: twilight annotation, calibration, location estimation, re-calibration, location re-estimation, and stationary period refinement [44, 55].

### Twilight annotation

Annotating twilight events is the basis for estimating locations using ambient light. A twilight event is calculated when the recorded light level crosses a chosen threshold. We defined twilights using a threshold of 3 lx (Mk18) or 2 lx (W65, C65K, and MK5090), which was above the background noise of the data and therefore avoided the inaccuracy of the sensor creating twilights. We then removed outlier twilight events that were greater than 45 min different from the surrounding twilights within a 4-day window, following guidelines for the *FLightR* package [54].

### Calibration and location estimation

Calibrating the data was an essential step in converting ambient light into location estimates [44], because geolocators varied in precision and used differing measurement scales for ambient light depending on the manufacturer. Calibration involved fitting a log-linear relationship between expected and observed light levels at a known location and time where the animal remained stationary for several weeks. Because the Arctic breeding site in our study was above the Arctic Circle (66.7° N), where there are 24 h of daylight during summer, there were not enough detectable twilights available for calibration. To compensate, we used a two-step calibration process that involved creating a preliminary calibration using the individual’s banding site and Izembek as known summer and fall locations, identifying approximate stationary locations from this preliminary calibration, then re-calibrating the data using the longest stationary location from each bird’s preliminary migration track as a known location [54] before deriving final location estimates.

After each calibration step, we calculated twice-daily location estimates using a state-space hidden Markov model [56] informed by annotated twilights, calibration output, and a spatial mask [54] that defined the plausible limits of migration paths based the species’ known range and habitat preferences. We constrained stationary locations to be within 20 km of the coast based on the preference of brant for nearshore eelgrass habitat, but we allowed individuals to travel up to 500 km offshore to account for long-distance flights, which we restricted to a maximum of 1700 km between twilights [57]. We then identified all stationary locations where birds remained stationary for ≥ 4 days.

In cases where the longest stationary location from a preliminary migration track did not correspond to the winter season due to an extended fall stopover at Izembek, we used the second-longest stationary location for the final calibration. To prevent overfitting of the calibration model, we truncated stationary locations to be a maximum of 30 days. Once the two-step calibration process was complete, we used an ‘on-the-fly’ outlier detection algorithm to omit unrealistic location estimates [54]. We extracted stationary locations from the final migration track where the bird remained stationary for ≥ 4 days. For consistency, we applied the same multi-stage calibration process to Arctic- and subarctic-breeding brant.

### Stationary period refinement

The coarse spatial resolution of geolocators produces location estimates that are only accurate to approximately 250 km [58]. To accommodate this level of uncertainty, we merged stationary locations for each animal that were less than 250 km apart and used the mean latitude and longitude of the merged sites. Additionally, we omitted any stationary location less than 250 km from the breeding location or north of the Arctic Circle.

### Evaluating the migratory network

We aggregated individual migration tracks to create a migratory network. Four tags were retrieved several years after deployment and contained data spanning multiple annual cycles, but we only used the first year of data for these birds to avoid pseudoreplication. To assess the temporal component of migration behavior, we compared the dates when individuals left their breeding sites, their spring and fall stopovers at Izembek, and their wintering sites, and when they returned to the breeding site. To assess the spatial component, we calculated the migratory connectivity metric [59] using the R package *MigConnectivity* 0.4.2 [59], which quantifies the spatial overlap of two or more populations from one season to the next. This approach approximates the Mantel correlation, a common metric used to quantify connectivity [60] but improves upon it by incorporating error and bias into the analysis. We estimated migratory connectivity based on the variance, covariance, and bias of location estimates, the relative abundance at the breeding and wintering sites, and the probability of moving from each breeding site to each wintering site during an annual migration (which we refer to as ‘transition probabilities’ following the terminology used by authors of the *MigConnectivity* package [61]).

Typically, location uncertainty and directional bias are calculated when birds are at breeding sites [61]. Because the Arctic breeding site lacked twilight in summer, we calculated these parameters at Izembek instead. We used Izembek to estimate bias and uncertainty for both the Arctic and subarctic breeding populations to maintain a consistent approach. For each bird, we identified the fall stationary periods that were within 500 km of Izembek and merged them. We chose the size of the merge radius to account for the increased uncertainty of geolocators around the fall equinox. We then calculated the minimum arrival date, maximum departure date, mean latitude, and mean longitude for all merged stationary periods. Then, we isolated all daily locations between the minimum arrival and the maximum departure of the fall stopover to get daily location estimates at Izembek for each bird. We treated the centroid of Izembek Lagoon as the actual location, and then we calculated the difference between estimated and actual locations as our measure of error. We determined directional bias as the mean distance between estimated locations and Izembek, and quantified location uncertainty using the variance and covariance of latitude and longitude estimates during the fall stationary period at Izembek.

To identify potential wintering sites, we used known eelgrass beds along the Pacific coast of North America [62, 63] and averaged the coordinates among eelgrass bed locations less than 250 km apart (Table 1; Fig. 1). We then identified the southernmost stationary location identified for each brant (hereafter, target points), and assigned winter site affiliations for all brant by determining the closest potential wintering site to each target point. We assumed the actual locations of the target points were likely to be within one of the wintering sites due to brant’s reliance on eelgrass, and the patchy distribution of eelgrass habitat across their migratory range.

**Table 1:** Potential wintering sites of geolocator-tagged Pacific black brant (*Branta bernicla nigricans*) containing eelgrass (Zostera spp.)

Wintering site	Eelgrass beds	Latitude	Longitude
Izembek	Izembek Lagoon	55.270	− 162.910
British Columbia (BC)	Skidegate Inlet and Masset Inlet	53.28	− 132.095
Washington/British Columbia (WA/BC)	Dungeness Bay, Puget Sound, Fidalgo Bay, Padilla Bay, Samish Bay, Bellingham Bay, Lummi Bay, Birch Bay, Boundary Bay, Roberts Bank, Sooke Basin, Saanich Peninsula, Victoria Peninsula, Parksville-Qualicum Beach, Baynes Sound-Comox Harbor, and Campbell River area	48.560	− 123.049
Oregon/Washington (OR/WA)	Coos, Yaquina, Tillamook, Netarts, Nehalem, and Willapa Bays, and Grays Harbor	45.201	− 123.796
North California (North CA)	Humboldt Bay	40.410	− 124.130
Central California (Central CA)	Bolinas Lagoon, and Drakes, Tomales, and Bodega Bays	37.963	− 122.495
South California (South CA)	Morro Bay, San Diego, and Mission Bay	35.210	− 120.500
Baja California, Mexico (Mexico)	Embayments of Baja California, Sonora and Sinaloa	27.580	− 113.990

Potential wintering sites containing eelgrass beds within the winter range of black brant (brant) were identified from the literature [62, 63]. To characterize the winter distribution of brant, eelgrass beds less than 250 km apart were aggregated into regional wintering sites, based on previous estimates of location uncertainty for geolocators

To calculate transition probabilities between each breeding site and each winter site, we used the estTransition function from the package *MigConnectivity* [59]. These transition probabilities, along with the relative abundances of the Arctic and subarctic breeding populations and the distance between breeding sites, allowed us to estimate migratory connectivity between the two populations. Migratory connectivity values range from − 1 to 1, where values close to 0 indicate that breeding populations mix freely during migration and at overwintering sites. Negative values indicate that individuals which are close in the breeding season are farther apart in the fall and winter, or vice versa, while positive values indicate that populations are separated into distinct flyways throughout their migration [59].

Transition probability estimates could have been biased by unequal sampling probabilities between sites. To reduce this bias, we used counts from the U.S. Fish and Wildlife Service Mid-Winter Waterfowl Survey, averaged from 2011 to 2014 [10], to estimate the relative abundance of brant at each winter site in our study. The Mid-Winter Surveys report state-level abundances, which we partitioned among potential eelgrass-containing winter sites based on published estimates of peak spring brant abundance for all winter sites [63]. We then divided by the total mid-winter count to calculate relative abundances for all potential winter sites. The mid-winter survey almost certainly undercounts brant because the total is < 60% of recent photo surveys of the fall staging population at Izembek Lagoon [10, 64], but it is the best available long-term record of brant relative abundance across their winter range in North America. To calculate relative abundances at the breeding sites, we divided estimates of breeding abundance in the Arctic [33] and subarctic [34, 65] by their sum.

To compare differences between the two breeding populations in the temporal use of sites within the migratory network, we calculated seasonal departure and arrival dates for breeding sites, wintering sites, and fall/spring migration stopovers at Izembek. We defined the start of fall migration as when brant were first ≥ 250 km from their respective breeding site. However, we removed individuals that wintered at Izembek from the analysis of fall departure. We used the same date ranges for the wintering period that we calculated for the migratory connectivity metric. For the northward spring migration, we aggregated any stationary periods that were ≤ 500 km from Izembek between March and May. We used a buffer larger than the expected 250 km location uncertainty for geolocators, to account for the greater uncertainty associated with the spring equinox. For all estimated locations that were aggregates of multiple stationary periods, we used the minimum arrival and the maximum departure dates. To calculate date of arrival at breeding sites, we removed any birds that did not record a complete migration cycle. For subarctic birds, we defined arrival at the breeding site as the earliest date when they were ≤ 250 km from the Tutakoke River (61.3° N, 165.6° W). This method did not work effectively for Arctic-breeding birds because there is a lack of twilight as the birds return to the breeding grounds. Therefore, we defined arrival to the breeding site for Arctic birds as the date when they crossed the Arctic Circle. We believe this to be a reasonable assumption given that brant can travel between the Arctic Circle and their Arctic breeding site in less than a day [57] and estimated arrival dates were comparable to field observations of first arrival dates on the Colville River Delta [66, 67].

To test for temporal differences in site use across our migratory network, we fitted Bayesian linear mixed models to test the effects of breeding site, wintering site, and breeding*wintering site interaction on seasonal arrival and departure dates, with a random effect of year to account for interannual variation in seasonal phenology. We specified uninformative normal priors for model coefficients, with a mean of 0 and SD of 10. For all analyses, we used JAGS version 4.3.0 and the runjags R package [68]. We ran each model with three chains of 1000 iterations and a 1000-iteration burn-in period to achieve convergence. We evaluated model convergence based on visual evidence of chain mixing and Gelman-Rubin statistic values < 1.1 for all parameters [69]. We also evaluated parameter identifiability by ensuring that the overlap between posterior and prior distributions was < 35% for all parameters [70]. We evaluated differences among breeding/wintering site combinations by comparing posterior mean estimates and 95% Credible Intervals, and by calculating the posterior probability that model parameters for breeding site, wintering site and interaction effects were not equal to 0.

### Nest phenology and survival

Most geolocator-tagged birds that nested following a complete migration were not physically monitored during the nesting period. Instead, we estimated the start of nest incubation and probability of nest success using light-level data. First, we identified non-nesting individuals by calculating the average noon light value during the beginning of the breeding season in May and June. Birds with an average noon light value exceeding a threshold of 45 lx were determined to be non-nesters. For those with light levels below 45 lx, we calculated the 1-week rolling average of mean noon brightness, and the first day that did not record a mean noon brightness of 64 lx was designated as the start of incubation. We verified this method by comparing against plots of lux versus time, in which the incubation period could be readily identified despite light-level variation due to incubation breaks [26]. We used the same threshold to identify the end of the incubation period, except when birds were captured on the nest to retrieve their geolocators.

After determining incubation start and end dates, we created daily encounter histories for each nest and estimated daily nest survival rates in a Bayesian framework. We could not determine nest fate directly from light-level data. Instead, we assumed that nests were successful if the incubation period was at least 24 days [71]. We used estimated start and end dates, along with assigned nest fates, to create encounter histories where daily nest status was coded as 1 (active), 0 (inactive), or NA (unknown). Encounter histories were right-censored (status = NA) if geolocators were retrieved via nest capture without subsequent nest monitoring and data collection ended while the bird was still incubating. We then modeled daily survival as a Bernoulli process $\left( {Nest\;status_{i,t + 1} \sim Bernoulli\left( {\varphi_{i} \cdot Nest\;status_{i,t} } \right)} \right)$, where φ_i_ = daily survival probability for nest *i,* and fit a linear model of daily nest survival as a function of breeding site, wintering site, and breeding*wintering interaction on the logit scale. After estimating daily nest survival, we estimated the probability of nest success by raising posterior daily nest survival estimates to the 24th power. We assigned the same uninformative priors that we used for models of migration arrival and departure dates and used the same methods to evaluate model convergence and compare posterior estimates between sites.

## Results

We attached 164 geolocators to brant from 2011 to 2014 of which 63 were retrieved from the two breeding locations. From this sample, we created a migratory network using 32 tags from the Arctic and 31 from the subarctic. Of these 63 geolocators, 57 of them recorded at least one complete migration cycle, which we used in our nest survival analysis.

**Fig. 2:**
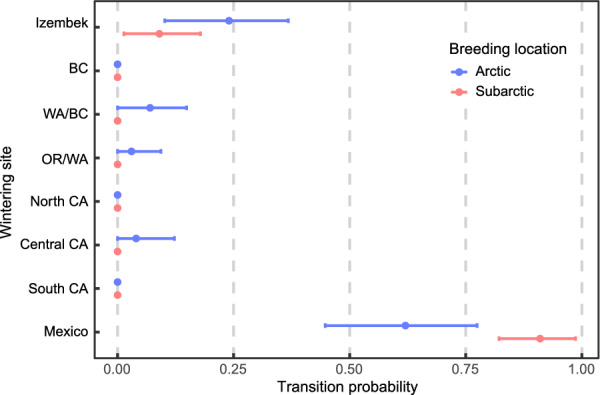
Transition probabilities for Pacific black brant (*Branta bernicla nigricans*) from breeding to wintering sites. Error bars around mean probability points are the 95% credible intervals. Transition probabilities represent the relative probability of movement between a breeding and a wintering site and sum to one across all possible wintering sites for each breeding site. Transition probabilities were estimated based on location estimates derived from light-level geolocators (n = 63). Brant were tagged with geolocators at a subarctic breeding site (Tutakoke River, Yukon Kuskokwim Delta National Wildlife Refuge; 61.3° N, 165.6° W) and an Arctic breeding site (Colville River Delta; 70.42° N, 150.38° W) in Alaska, USA. Wintering site locations are abbreviated as Izembek = Izembek National Wildlife Refuge, WA = Washington, BC = British Columbia, CA = California, Mexico = Baja California, Mexico

Arctic and subarctic breeding populations were well mixed during the winter (migratory connectivity = − 0.06, standard error = 0.03). Mexico was the most common wintering site for brant from either breeding location (Fig. 1). Of those that did not migrate to Mexico, most migrated to Izembek, and only four geolocators were tracked to wintering sites in British Columbia or the continental United States (Fig. 1). Despite these similarities between breeding sites, breeding site—wintering site transition probabilities revealed that subarctic birds were more likely to winter in Mexico (transition probability = 0.91) than those from the Arctic (transition probability = 0.62), and posterior credible intervals for each breeding site did not overlap with the posterior mean for the other site (Fig. 2). In contrast, Arctic birds were more than twice as likely to overwinter in Izembek (transition probability = 0.23) than subarctic birds (transition probability = 0.09), and once again the credible intervals for Arctic brant did not overlap with the posterior mean for the subarctic, and vice versa (Fig. 2). The two breeding populations began their fall migration at different times but synchronized their phenology at Izembek. Subarctic birds started their south migration on average 14 days earlier than the Arctic birds (*p*(breeding site difference > 0) > 0.999) and arrived at Izembek on average 12 days earlier than the Arctic birds (*p*(> 0) = 0.99). Excluding brant that remained to winter at Izembek, individuals from both breeding populations which continued their southward migration all departed within a few days of each other (Table 2, *p*(breeding site difference > 0) = 0.74).

**Table 2:** Migration phenology of Pacific black brant (*Branta bernicla nigricans*) based on light-level geolocators

Season	Wintering site	Event	Arctic-breeding	n	Subarctic-breeding	n
Breeding	–	Departure	Aug 15 (Aug 6–Aug 24)	33	Aug 1 (Jul 22–Aug 10)	31
Fall (Izembek)	–	Arrival	Sep 23 (Sep 14–Oct 3)	33	Sep 11 (Sep 2–Sep 21)	31
		Departure	Oct 20 (Oct 9–Oct 30)	21	Oct 16 (Oct 7–Oct 26)	27
Winter	Izembek	Arrival	Sep 13 (Sep 5–Sep 20)	9	Sep 6 (Aug 23–Sep 19)	3
		Departure	May 13 (Apr 27–May 30)	8	Apr 29 (Apr 5–May 25)	3
	Mexico	Arrival	Nov 19 (Nov 13–Nov 25)	21	Nov 14 (Nov 8–Nov 19)	27
		Departure	Mar 14 (Feb 28–Mar 27)	20	Mar 22 (Mar 11–Apr 2)	27
Spring (Izembek)	Mexico	Arrival	May 5 (Apr 28–May 11)	16	Apr 23 (Apr 18–Apr 28)	21
		Departure	May 31 (May 27–Jun 4)	16	May 22 (May 19–May 25)	21
Breeding	Izembek	Arrival	May 28 (May 21–Jun 4)	7	May 17 (May 6–May 29)	3
		Incubation	Jun 10 (Jun 6–Jun 14)	8	May 28 (May 20–Jun 4)	2
	Mexico	Arrival	Jun 1 (May 27–Jun 8)	16	May 19 (May 14–May 24)	27
		Incubation	Jun 10 (Jun 7–Jun 13)	11	Jun 1 (May 30–Jun 4)	23

Median departure and arrival dates at breeding sites, wintering sites, and fall/spring migratory stopovers for black brant (brant) from subarctic (Tutakoke River; 61.3° N, 165.6° W) and Arctic breeding sites (Colville River Delta; 70.42° N, 150.38° W) in Alaska, USA. Izembek Lagoon (Izembek) is used as a fall staging area by most brant, but it is also used as a wintering site or spring staging area. Winter arrival and departure dates are also shown for wintering habitat in Baja California, Mexico (Mexico). Brant that overwintered at Izembek were not used to calculate fall departure dates or spring arrival dates for Izembek. Arrival and departure dates were estimated using Bayesian mixed models with fixed effects: wintering site*breeding site and a random effect of year. 95% posterior credible intervals are in parentheses. n = sample size

Brant that remained at Izembek to overwinter arrived there approximately 3 weeks earlier than the birds that only stopped there in the fall and ended their fall migration 2 months earlier than birds that continued to wintering areas in Mexico (Table 2, *p*(> 0) = 1). Similarly, birds wintering in Mexico initiated their northward spring migration on average more than a month earlier than birds wintering in Alaska (Table 2, *p*(> 0) = 1). There was no support for an effect of breeding site affiliation on winter site arrival (*p*(> 0) = 0.93) and departure (*p* > 0) = 0.89) dates, although on average subarctic birds arrived and departed several days earlier than Arctic birds (Table 2).

Of the 48 geolocator brant that wintered outside of Alaska, 37 (77%) returned to Izembek in spring. Of these birds, subarctic birds arrived at Izembek in spring 11 days earlier than Arctic birds and departed for their breeding location 9 days earlier. These differences in spring arrival and departure days between breeding populations were strongly supported during spring staging at Izembek (*p*(> 0) = 1). Similarly, subarctic birds returned to their breeding site earlier than Arctic birds regardless of wintering site; more than 2 weeks earlier than Arctic birds on average when comparing brant that wintered in Izembek, and 8 days earlier for those that wintered in Mexico (*p*(breeding site difference > 0) = 0.98). Brant that used Izembek as a spring staging area departed later than brant which overwintered there on average, but some brant flew from Mexico back to their breeding sites without any identifiable stopovers, and when non-staging brant were included winter site choice (Izembek versus Mexico) did not have a strong effect on the date of spring arrival at either breeding site (*p*(winter site difference > 0 = 0.26); *p*(breeding site*wintering site interaction > 0 = 0.84); Table 2).

**Fig. 3:**
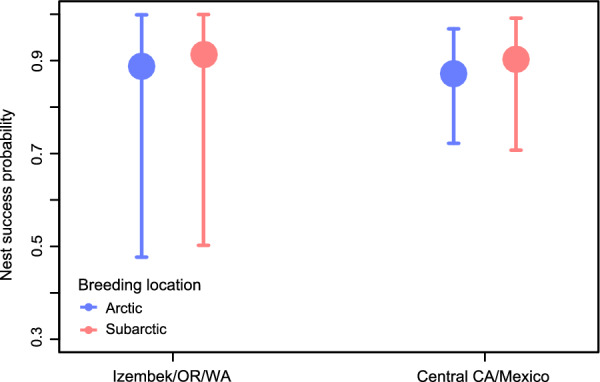
Probability of nest success for Arctic- and subarctic-breeding Pacific black brant (*Branta bernicla nigricans*) from different wintering sites. The probability of nest success was estimated based on incubation periods derived from light-level geolocators (n = 46). Nest success was estimated as daily nest survival probability^24^, based on an average incubation period of 24 days. Nest success was estimated separately for all wintering sites in the northern half of the non-breeding range, including Izembek Lagoon, and sites in Oregon and Washington (Izembek/OR/WA), and for the southern half of the nonbreeding range including central California and Baja California, Mexico (Central CA/Mexico). Other wintering sites in Table 1 were not included because no nesting data were available for brant associated with those wintering sites. Brant were tagged with geolocators at a subarctic breeding site (Tutakoke River, Yukon Kuskokwim Delta National Wildlife Refuge; 61.3° N, 165.6° W) and an Arctic breeding site (Colville River Delta; 70.42° N, 150.38° W) in Alaska, USA

Both breeding populations showed high nest survival regardless of their wintering site. Despite small sample sizes for some breeding/wintering site combinations, all nest survival model parameters showed good convergence and were identifiable, with < 1% overlap between posterior and prior distributions. The mean probability of a nest surviving until 24 days was between 0.88 and 0.92 for all breeding site/wintering site combinations, with substantial overlap among 95% credible intervals (Fig. 3). Our results indicated a high overall probability of nest success for individuals from the southern and northern extremes of the wintering range.

## Discussion

Our analysis of migratory connectivity between Arctic and subarctic brant provided new insights into recent changes in their winter distribution and the implications of those changes for individual reproductive success and population dynamics of brant in Alaska. Compared to previous studies, our analysis revealed a long-term trend towards earlier spring arrival dates at each breeding site [66, 72] and earlier fall departure dates from the subarctic [72]. These shifts were associated with earlier snowmelt and vegetative growth at high latitudes and mirror patterns documented in many other long-distance migrants [22, 66, 73]. While our analysis had limited power to detect differences in phenology and nest survival, our dataset appears to be representative of the Arctic and subarctic breeding populations when compared to previous studies [49, 67, 74]. We found that most birds from both breeding populations still wintered in Mexico, but subarctic birds were about 1.5 times more likely to winter there compared to Arctic birds. Therefore, recent declines in habitat conditions in Mexico have most likely had a greater impact on the subarctic breeding population of brant. In contrast, Arctic birds were twice as likely to winter at Izembek. This is consistent with the distribution of band recoveries from Izembek [11] and indicates that the growing Arctic population is disproportionately responsible for the northward shift in the wintering brant population [22]. Lastly, we did not find support for the hypothesis that remaining in Alaska year-round was an adaptive behavior. Specifically, there were no differences in nesting phenology or nest success between birds wintering in Alaska and Mexico. Our results showed that Arctic and subarctic breeding populations were strongly connected across their winter range, but the continued growth of the Arctic breeding population and increased winter use of Izembek are unlikely to offset observed declines in the subarctic breeding population, given that the current size of the Arctic breeding population is six times smaller than the subarctic.

Arctic- and subarctic-breeding brant arrived at their breeding grounds and departed for fall-staging areas earlier than in previous decades. In our study, 95% of the subarctic birds arrived at the breeding site on the Yukon-Kuskokwim Delta before first arrival of radio-tagged birds arrived there approximately a decade earlier in 2000 [72]. In Arctic Alaska, brant have also been steadily advancing their arrival to nesting areas on the Colville River Delta [66]. Examples of long-distance migrants returning earlier to their breeding grounds are widespread in the literature (e.g. [73]) and are consistent with long-term warming trends leading to earlier spring conditions at brant breeding areas in Alaska [66]. We also found that 95% of the subarctic brant in our study departed from their breeding site earlier than the mean departure date observed in 2000 [72]. These earlier fall departures are likely a reflection of the advancing nesting phenology observed in the Arctic, which would allow goslings to fledge earlier in the year.

Small sample sizes limited precision in our estimates of demographic rates for some combinations of breeding and wintering sites, but nevertheless our geolocator dataset appeared to be representative of the two breeding populations based on comparisons to previous research. For example, estimates of the mean date of incubation onset for Arctic birds were within 2 days of estimates derived from a nest monitoring dataset with hundreds of nest records during the same period [49, 67]. Our nest survival estimates may have been biased high compared to traditional nest monitoring methods, because we may have failed to detect some instances of late nest failure or nests that failed to hatch but were not abandoned. On the other hand, geolocator-based nest survival estimates may have been less biased because each nest could be monitored starting on day one of the incubation period, and nests were not periodically disturbed so daily nest survival estimates did not need to account for negative ‘observer’ effects. Although we did not have comparable estimates for the timing of incubation for the subarctic birds, the subarctic credible intervals for our estimates of nest survival probability overlapped with estimates from nest monitoring plots at the breeding site in 2012 and 2013, which were two of the latest nesting years on record for brant on the Yukon-Kuskokwim Delta [74].

The majority of Arctic and subarctic brant wintered in Mexico, and as such, both breeding populations were susceptible to changing habitat conditions there. Bahia San Quintín, a protected bay in Baja California, Mexico that has historically served as the primary wintering area for brant [24] has suffered a long-term decline in eelgrass abundance [17, 75]. This reduction in habitat quality at a shared winter site may help explain why overwinter survival rates have declined recently in both breeding populations [76]. Increasing ocean temperatures due to climate change [77] will likely exacerbate the decline in eelgrass by reducing net primary productivity and causing more frequent die-offs [78]. Since brant feed almost exclusively on eelgrass during the non-breeding season [75], these declines may continue to have negative carry-over effects on survival and fecundity for brant nesting in Arctic Alaska as well as the subarctic [12, 23]. Subarctic birds, however, were 1.5 times more likely to winter in Mexico than Arctic birds (Fig. 2). If this pattern continues, the consequences of future eelgrass losses in Mexico will probably be more severe for the declining subarctic breeding population.

For brant that did not go to Mexico, the overwhelming majority stayed at Izembek, and only a few individuals used eelgrass beds in Canada or the continental United States. Arctic-breeding brant were more than twice as likely as subarctic breeders to use their northernmost wintering location at Izembek. Our findings were consistent with band recoveries from 2010 – 2016, which indicated that all brant that were harvested and reported during the winter at Izembek had been banded in the Arctic [11]. Subarctic birds, however, probably still comprised most breeding adults in the winter population at Izembek, because the subarctic breeding population remains six times as large as the Arctic population based on a recent literature review [36]. Although brant from both breeding sites returned to traditional wintering areas in Mexico, the majority also stopped in Izembek for 1–2 weeks on their return journey in the spring. Izembek Lagoon and nearby eelgrass coastal areas are well known for their critical importance to brant during fall migration, but they were also heavily used throughout the winter and spring [17].

Despite shortening their migration by thousands of kilometers, brant that overwintered at Izembek did not appear to derive a clear reproductive advantage. Brant returning from wintering sites in Alaska and Mexico reached their breeding areas and began incubation at similar times, and overwintering in Izembek was not associated with an increase in nest success. The energetic gain from a shorter migration may have been equivalent to the energetic costs associated with spending the winter in Alaska instead of Mexico [12], and the use of Izembek as a spring migration stop could have allowed brant returning from Mexico to recover some of the energetic reserves depleted by their long migration. Even so, wintering near breeding sites is typically expected to improve reproductive success by allowing migrants to arrive earlier and increase synchrony between the timing of their arrival and spring weather conditions [79, 80], but our study did not support that hypothesis as an explanation for the migratory movements of brant. Overwintering in Alaska could provide a more definitive advantage for Western High Arctic brant, or for black brant from high arctic breeding sites in Russia and Canada, since these populations must travel hundreds of kilometers farther to reach Izembek and their breeding areas. Incorporating these groups into future migratory connectivity studies could paint a more complete picture of brant metapopulation dynamics and shed further light on the mechanisms responsible for their shifting winter distribution.

We were not able to test whether wintering at Izembek affected other components of reproduction like clutch size or breeding propensity, but the rapid increase in the winter population at Izembek has coincided with a long-term decline in brant recruitment [81, 82]. Since we conducted our study, the nesting population of brant and the availability of grazing lawn habitat on the Yukon-Kuskokwim Delta have continued to decline [10, 83] while Arctic sites have continued to provide abundant brood-rearing habitat [84]. The winter population at Izembek has also continued to grow, and even exceeded the winter population in Mexico in 2023 [10], but this increase cannot be explained solely by recruitment from the Arctic. The winter shift towards Izembek could have been driven mainly by young pre-breeding adults, which are often responsible for migratory innovations in geese [85], but the concurrent decline in overall recruitment makes that explanation less plausible [81]. It could also be partly due to changes in the winter distribution of non-breeding adults and failed breeders, including brant from the Western High Arctic population [57]. Even for the two breeding populations in this study, brant that overwintered at Izembek arrived weeks ahead of fall-staging individuals, which raises the possibility that overwintering birds were more likely to have lost their broods prior to fledging or during migration. Regardless, the Arctic breeding population of brant appears to have grown despite, rather than because of, their disproportionate use of a shorter migration strategy. Eelgrass abundance at Izembek was stable during the study period [86] but a more recent analysis identified patches where abundance has declined, which could further limit the energetic benefits of overwintering there [87]. Collectively, our results suggest that continued growth of the winter population at Izembek is unlikely to increase brant recruitment overall or compensate for the declining subarctic population, especially while a substantial fraction of both Alaska breeding populations continues to use degraded wintering habitat in Mexico.

The dramatic shift in the wintering distribution of brant [10] is an extreme example of how migratory species with specialist food preferences can respond to changes in habitat availability due to climate change [23]. Northward range shifts due to climate change have been widely documented, but these changes are typically a few kilometers per decade [73], several orders of magnitude smaller than the distance between the traditional wintering areas for brant in Mexico, and their more recent wintering site at Izembek Lagoon in Alaska. Because the Arctic is warming more rapidly than the rest of the world [88], new areas of suitable winter habitat are likely to emerge for other long-distance migrants that breed at high latitudes, but the largest winter range shifts may be more likely to occur in species, like brant, that are constrained by a scarce and patchily distributed resource. The demographic implications of such distributional shifts, however, are difficult to predict. For some Arctic-breeding migrants like common eiders, using more distant wintering areas means that individuals arrive later at their breeding areas, which can lead to later nest initiation and lower reproductive output [89]. The contrasting pattern displayed by brant suggests that migration distance alone is not a reliable predictor of breeding phenology or reproduction, especially for populations that use fall and spring staging areas where they can recoup energetic losses or wait out unfavorable conditions during migration.

## Data Availability

The datasets supporting the conclusions of this article are available in two online data releases [90, 91].

## Abbreviations

BC: British Columbia
CA: California
Izembek: Izembek Lagoon
geolocators: Light-level geolocators
OR: Oregon
brant: Pacific black brant, *Branta bernicla nigricans*
WA: Washington State
